# Prefrontal Asymmetry BCI Neurofeedback Datasets

**DOI:** 10.3389/fnins.2020.601402

**Published:** 2020-12-18

**Authors:** Fred Charles, Caio De Castro Martins, Marc Cavazza

**Affiliations:** ^1^Faculty of Science and Technology, Bournemouth University, Poole, United Kingdom; ^2^School of Computing and Mathematical Sciences, University of Greenwich, London, United Kingdom

**Keywords:** functional near infrared spectroscopy (fNIRS), PFC asymmetry, visual feedback (VF), neurofeedback (NF), dataset

## Abstract

Prefrontal cortex (PFC) asymmetry is an important marker in affective neuroscience and has attracted significant interest, having been associated with studies of motivation, eating behavior, empathy, risk propensity, and clinical depression. The data presented in this paper are the result of three different experiments using PFC asymmetry neurofeedback (NF) as a Brain-Computer Interface (BCI) paradigm, rather than a therapeutic mechanism aiming at long-term effects, using functional near-infrared spectroscopy (fNIRS) which is known to be particularly well-suited to the study of PFC asymmetry and is less sensitive to artifacts. From an experimental perspective the BCI context brings more emphasis on individual subjects' baselines, successful and sustained activation during epochs, and minimal training. The subject pool is also drawn from the general population, with less bias toward specific behavioral patterns, and no inclusion of any patient data. We accompany our datasets with a detailed description of data formats, experiment and protocol designs, as well as analysis of the individualized metrics for definitions of success scores based on baseline thresholds as well as reference tasks. The work presented in this paper is the result of several experiments in the domain of BCI where participants are interacting with continuous visual feedback following a real-time NF paradigm, arising from our long-standing research in the field of affective computing. We offer the community access to our fNIRS datasets from these experiments. We specifically provide data drawn from our empirical studies in the field of affective interactions with computer-generated narratives as well as interfacing with algorithms, such as heuristic search, which all provide a mechanism to improve the ability of the participants to engage in active BCI due to their realistic visual feedback. Beyond providing details of the methodologies used where participants received real-time NF of left-asymmetric increase in activation in their dorsolateral prefrontal cortex (DLPFC), we re-establish the need for carefully designing protocols to ensure the benefits of NF paradigm in BCI are enhanced by the ability of the real-time visual feedback to adapt to the individual responses of the participants. Individualized feedback is paramount to the success of NF in BCIs.

## 1. Introduction and Rationale

There is growing interest in sharing datasets for Brain-Computer Interfaces (BCI), to facilitate comparison of technical approaches. Their availability is of particular relevance for applications in which there is significant diversity of practice and lack of standardized protocols, such as Neurofeedback (NF) (Ros et al., 2020).

Such datasets make it possible to explore and compare signal acquisition and dynamics, baselining, thresholding, and categorization: this has the potential to identify experimental difficulties and best practice, beyond reproducibility issues.

Among the various neural signals that support BCI, the availability of fNIRS dataset remains scarce (Bak et al., 2019), despite its growing popularity, both for BCI (Naseer and Hong, 2015) and NF applications (Kohl et al., 2020).

In this paper, we introduce three datasets obtained as part of fNIRS BCI experiments. The originality of these datasets is that they were produced in a BCI context yet using a NF paradigm, in which users control their Prefrontal Cortex (PFC) asymmetry. The use of a NF approach to BCI is characterized by an emphasis on RoI activation over long-term effects, often with minimal training compared to clinical uses of NF. It is of particular interest when the RoI signal is not under direct volitional control, as the NF channel assists the user in controlling the signal. Moreover, the feedback channel can be embedded in the interface design itself for added realism. Frontal asymmetry is an important brain signal which has a long history in BCI, for the measure of valence, approach or cognitive workload, and NF. Since frontal signals are of the main elements of fNIRS, this dataset has validity beyond the specific context it has been produced in, which is PFC asymmetry NF.

After a reminder of key concepts in NF, which includes a short discussion of current thinking in fNIRS NF, we discuss the potential interest of our datasets to the wider fNIRS and NF community, and describe several data formats supported by our dataset to facilitate processing by various software packages and data-oriented programming languages. In the remainder of the text we will refer to our three datasets as follow:

- **ANG** (Aranyi et al., 2015b) is derived from an anger-expressing BCI experiment.
- **RAP** (Aranyi et al., 2016) investigates rapport with a virtual character endowed with full facial expressions.
- **HEU** (Cavazza et al., 2017) uses BCI input to a hybrid human-AI system.

### 1.1. PFC Asymmetry in Neuroscience Research

One of the major challenges for BCI is to relate neural signals to specific cognitive processes, or to an element of user experience. For affective BCI, this is rendered even more difficult by the weakness of locationist hypotheses (Lindquist et al., 2012). However, there is substantial evidence linking prefrontal cortex (PFC) asymmetry to the approach/withdrawal dimension (Davidson, 2004): the paradox being that an area associated to high-level integrative cognitive processes is also the locus of a rather basic dimension. This dimension has been shown to underpin higher-level behavioral elements including motivation, risk-taking, aggression, and empathy. Moreover, it has been associated to clinical conditions, such as addiction, eating disorders, gambling and depression. While PFC asymmetry has been primarily associated with approach/withdrawal it has also been shown to be highly correlated with valence, as well as cognitive workload.

Historically, interest in PFC asymmetry has stemmed from research in affective and social neuroscience. Another significant use of PFC asymmetry has been early NF experiments, primarily for the treatment of depression (Rosenfeld et al., 1995). PFC asymmetry has been later adopted as a BCI technology taking advantage of the above results, and has been used for affective computing (Mühl and Heylen, 2009) cognitive workload measurement (Fishburn et al., 2014; Peck et al., 2014; Barth et al., 2016; Maior et al., 2020) or assessment of aesthetic response (Karran et al., 2015; Cartocci et al., 2016).

Most of early work on PFC NF has taken place using EEG signals. There are several reasons for that: despite the lack of spatial resolution, it is still possible to capture a meaningful PFC asymmetry signal from *F3* and *F4* electrodes. The existence of a stable PFC asymmetry EEG baseline in the alpha spectrum and the trait and state properties of the signal (Coan and Allen, 2002) facilitates the design of PFC NF experiments. With the increasing availability of fNIRS equipment, it appeared as an interesting alternative to EEG with less sensitivity to a range of artifacts and increased specificity and spatial resolution considering that the RoI is close to the surface hence easily accessible to infrared sensors. Sitaram et al. (2009) were amongst the first to suggest that signals based on metabolic activity could be equally suited to BCI than electrical signals. fNIRS has become the method of choice as DLPFC is readily accessible via lateral optodes (Ernst et al., 2013). It has been used as a measurement tool (Hirshfield et al., 2007) and as a BCI (Solovey et al., 2009; Afergan et al., 2014; Naseer and Hong, 2015; Hong et al., 2020) to support NF research and even clinical experiments.

### 1.2. Neurofeedback Concepts

Our dataset has been entirely produced through NF BCI experiments and it is worth summarizing some of the main concepts attached to NF technical implementations and subject behavior. In naïve terms, NF consists in facilitating the activation of a region of interest in the subject's brain through the real-time display of a feedback signal that represents how successful they are in activating that region^1^. NF is generally considered an operant conditioning mechanism, and subjects tend to develop or improve this ability through training, although this ability shows great individual variability, some subjects demonstrating it from the very first testing sessions while others hardly develop it, a phenomenon close to BCI illiteracy (Lee et al., 2014; Trambaiolli et al., 2018).

The essential components of a NF installation (Sitaram et al., 2017) include a sensing device (EEG, fNIRS, FMRI, MEG) that captures a signal measuring the RoI activity, a software component analysing NF performance (by comparing the RoI signal to a baseline or reference), and a feedback system which maps the performance measure to a feedback channel giving the user an indication on how well they are activating the target region.

If we leave aside the case of motor areas, most NF experiments require the activation of areas which are not under direct volitional control^2^ [for instance, the amydgala (Zotev et al., 2016), insula (Lawrence et al., 2014), PFC (Barth et al., 2016), Anterior Cingulate Cortex (Mathiak et al., 2015; Zilverstand et al., 2017)]. Initially, subjects may use cognitive strategies to facilitate the activation of the target RoI and subsequently guide themselves on the feedback signal to sustain that activation. A cognitive strategy is essentially a set of thought contents which are known to facilitate the activation of the RoI, albeit not always specifically. For instance, imagining a gesture would activate corresponding pre-motor areas, pleasant autobiographic memories can affect PFC asymmetry, concentrating on your inner self may activate the insula (Lawrence et al., 2014), and achieving a relaxed state may decrease the amygdala activity. Cognitive strategies may be suggested by the experimenter, or may be discovered by the subjects themselves based on the (often partial) experimental brief they have been given (Autenrieth et al., 2020). Some of the cognitive strategies may actually lack specificity: for PFC asymmetry in which valence and approach may be confounded, the use of positive autobiographic memories as a cognitive strategy may actually bias activation from a valence perspective. Similarly, different strategies may lead to the same region activation (Lawrence et al., 2014).

Barth et al. (2016) have identified no less than 17 different cognitive strategies used by subjects during a PFC NF experiment (not involving PFC asymmetry). These were often workload-oriented and included verbal fluency tasks, calculating, and naming terms in certain categories. Only two subjects used emotional or arousing strategies, probably due to the fact that there were no affective or motivational element in that PFC experiment: subjects were given visual feedback of their own PFC through an activation heat map, and told to increase activation. Another possible explanation is that subjects may have been influenced in their choice of cognitive strategies by a preliminary working memory task undertaken prior to the actual experiment.

The cognitive strategies adopted by our subjects are specific to each experiment and, short of being dictated by the experimenter, were influenced by the context of the experiment and some high-level instructions given. In the ANG dataset, subjects naturally expressed anger at the designated character, while in the RAP dataset they tried various positive mental attitudes toward the agent. Finally, in the HEU dataset, they had to express motivation or eagerness and developed various strategies, such as mentally encouraging participants in a race.

The NF loop operates by measuring the level of activation, and mapping it onto the feedback signal, so that it reflects in real-time how successful the subject is in activating the RoI. The NF literature, despite its abundance, rarely discusses in-depth this mapping process/function, which is in general a linear mapping between the activation range above the baseline to the variation range of visual feedback. For instance, in our ANG experiment we run preliminary experiments measuring signal variation and define a variation range using the PFC asymmetry signal's standard deviation, in a subject-specific fashion.

**Figure 1:**
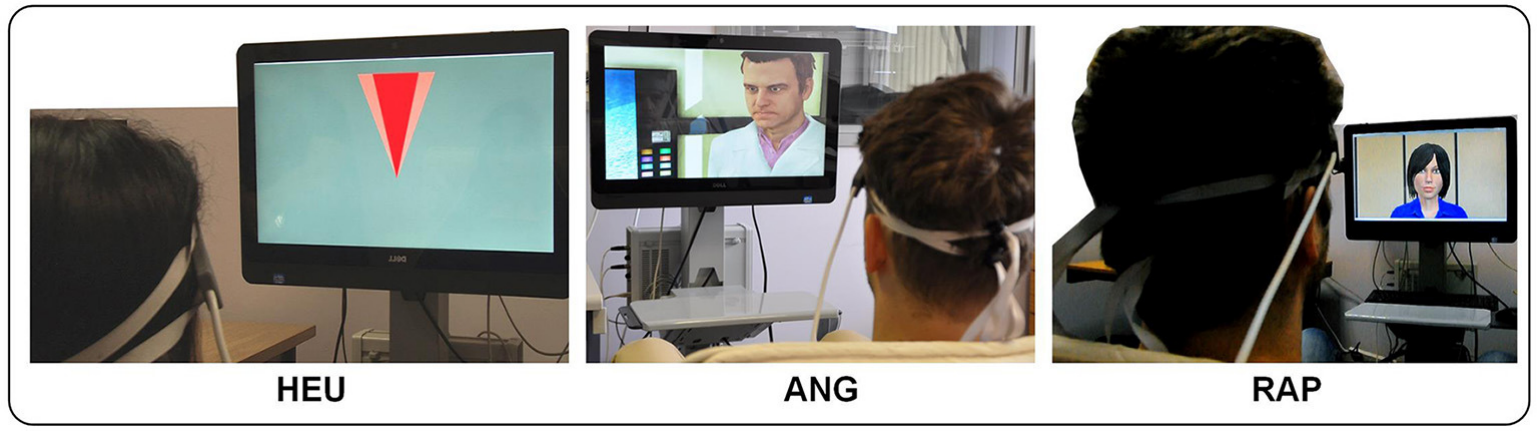
NF Feedback categorization: abstract [HEU], semantic [ANG], and task-related [RAP].

There has been growing interest in the nature of the NF feedback channel which can be acoustic (Rosenfeld et al., 1995), or more often, visual. In the latter case, the primary consideration is in the use of abstract symbology or visually realistic signal. To refine this distinction, we propose to categorize the type of visual feedback by taking into account its degree of integration with the interactive application controlled by the BCI (see Figure 1 for visual details).

**Abstract feedback** is the dominant modality in NF, and resorts to various gauges or abstract geometrical shapes whose size vary with the activation signal (Trambaiolli et al., 2018), or even screen color (Sakatani et al., 2013). Some feedback can be visually realistic but not semantically related, as in Li et al. (2019). Even feedback based of visualizing target brain areas as in (Barth et al., 2016) should fall in this category. Abstract feedback is primarily used in clinical applications or fundamental NF investigation: in BCI, abstract symbology tends to be disconnected from the main application, unless some metaphor can be established between the abstract shape and an element of the application. For instance, in our HEU dataset, the width of the triangle used for visual feedback is a metaphor of the heuristic search space (Cavazza et al., 2017).

**Semantic feedback** corresponds to more realistic visual feedback which can relate to the affective signals captured by the BCI signal. For instance, in our ANG dataset visual feedback consists in altering the visibility of the character against which anger has to be expressed after an animation showing his evil nature is shown to the subject.

Finally, **Task-related feedback** refers to experimental conditions in which the BCI input is naturally embedded in the interaction process, for instance with a visual feedback which is part of the interface operation rather than added-on symbology. In our RAP dataset, the overall task consists in non-verbal communication with an agent with the agent's non-verbal behavior actually constituting the feedback signal, making the feedback signal indistinguishable from the task itself. This comes at the cost of losing some real-time properties of the feedback signal, but the benefits of visual realism may actually outweigh this loss by far. In previous work with a different yet compatible signal [fMRI-derived EEG known as electrical fingerprint (Keynan et al., 2019)], we have suggested that some complex visual interfaces may have signal filtering abilities (Yamin et al., 2017).

While visually realistic feedback has been shown to facilitate NF performance via increased competence rather than simple engagement (Cohen et al., 2016) there is no evidence that it would distort the process compared to abstract feedback, which is why we consider our datasets as representative examples of generic interest. Moreover, visually realistic feedback with a social component (such as in our RAP dataset) has been shown to foster good NF responses even with minimal training (Mathiak et al., 2015). Finally, more speculative explanations could involve improved reward encoding with realistic visual feedback, in some cases even resonating with reward encoding in the RoI itself (Cavazza, 2018), in particular in the case of DLPFC (Tanaka et al., 2006; Aupperle et al., 2015).

NF signals tend to fluctuate significantly during an epoch. There have been several theoretical hypotheses underlying their dynamics, such as the difficulty to activate the region, the difficulty to sustain that activation, and the extent to which feedback could assist or even hinder the process (Hinterberger et al., 2004). Some researchers have hypothesized a control theory model for NF (Ros et al., 2014), in which oscillations would be explained by the response of the controller outside of a steady state mode. BCI uses of NF signal can operate with shorter epochs as no long-term effects are sought, and the actual duration tends to be a compromise between application requirements and signal acquisition.

## 2. Origins of Data

### 2.1. Common Description of fNIRS NF Experiments

Based on previous literature (Ruocco et al., 2014), including literature applying HbO to affect-related manipulation in the DLPFC (Tuscan et al., 2013), and to approach/withdrawal-related experimental manipulation (Morinaga et al., 2007), and based on our pilot study (Aranyi et al., 2015a), we elected to use HbO for real-time application; we based *post-hoc* analyses on the same metric for consistency.

Note that this measure is relative to a *baseline* (Ayaz et al., 2010), this has important practical consequences in defining and quantifying NF success. For example, as this operationalization of asymmetry yields interval-level data, a ratio of task/no-task signals for defining and quantifying success (for instance Sarkheil et al., 2015) cannot be applied.

In our previous work corresponding to the three datasets ANG, RAP, HEU, we have used a specific terminology in which *baseline* referred to signal value at rest. In some experiments, we used a reference epoch to calculate the signal variation, which in some instances required the subject to watch a similar environment to the one used during NF epochs, sometimes also involving a neutral cognitive task, such as *counting*. It should also be noted that some datasets have considered PFC asymmetry to be zero for the baseline whilst there is evidence of default PFC asymmetry values even in fNIRS (Zohdi et al., 2020) something which was readily captured in EEG experiments (Cavazza et al., 2014) but needs to be redefined on a session or even epoch basis when using hemodynamic signals.

In all these experiments NF is used for its ability to produce a signal with a clear interpretation in cognitive terms. We are using PFC asymmetry as a dimensional marker of approach, the actual cognitive feature under consideration (or analysis) being determined by the experimental context, and the nature of the feedback signal. For instance, an experiment on anger will measure approach (dissociated from valence) (Harmon-Jones, 2007), while in an experiment on empathy, approach can be used as a proxy measurement (Cavazza et al., 2014).

It is worth discussing again the main differences between clinical NF and BCI NF, the latter still being an emerging application within the broader field of BCI technology. In terms of experimental protocol and validation, clinical NF tends to rely on sham feedback as a control group, under the hypothesis that appropriate feedback provides the reward signal that mediates long-term effects. The clinical context implies and allows the use of repeated sessions with significant training, which increases the number of responders: on the other hand, BCI NF dedicates limited time to subjects training and leaves non-responders to the various categories of BCI illiteracy (Lee et al., 2014; Trambaiolli et al., 2018; Autenrieth et al., 2020), concentrating instead on the responders' behavior.

### 2.2. Representativity and Interest of the Dataset

In this section, we are briefly discussing the relevance and potential community interest of our datasets, considering the increasing popularity of fNIRS and in particular fNIRS NF. We will be basing this discussion primarily on the recent review of fNIRS NF by Kohl et al. (2020), which collected a significant number of studies and highlights variants in different core aspects of NF^3^.

Firstly, most of the studies reported in the review use HbO, which is also the case for our three datasets (Aranyi et al., 2015a). Recently (Tachtsidis and Scholkmann, 2016) have suggested that HbO alone might be insufficient to cover the widest range of experimental situation, but this recent observation has not yet been fully taken up in the community.

The duration of NF epoch in Kohl et al. (2020) ranges from 5 to 40 s (the latter actually corresponding to our own RAP dataset, although its actual useful duration is 33 s), with the majority of epochs (30%) lasting 30 s. Duration of epochs in our datasets are 30 s [HEU], 15 s [ANG], and 33 s [RAP].

Our subject population was primarily drawn from healthy subjects, which were also overrepresented in Kohl et al. (2020)'s review (76%). Our target RoI also proves to be one of the most studied ones, as 59% of the studies reviewed trained participants to regulate parts of the PFC.

Our three datasets also cover a range of cognitive strategies, ranging from explicitly expressing a given feeling [ANG], engaging with a virtual character [RAP], or expressing motivation [HEU]. We collected post-experiment user feedback (Autenrieth et al., 2020) on the actual cognitive strategies they used for NF: it highlighted a mix of through contents related to both approach and positive valence, apparently influenced by the visual nature of the application. Users reported various sorts of mental “cheering” as if encouraging runners during a race, as well as the use of more abstract thinking strategies to generate a feeling of eagerness, such as reminiscence of appetitive stimuli or pleasant memories.

## 3. Detailed Experiments

### 3.1. Apparatus

**Figure 2:**
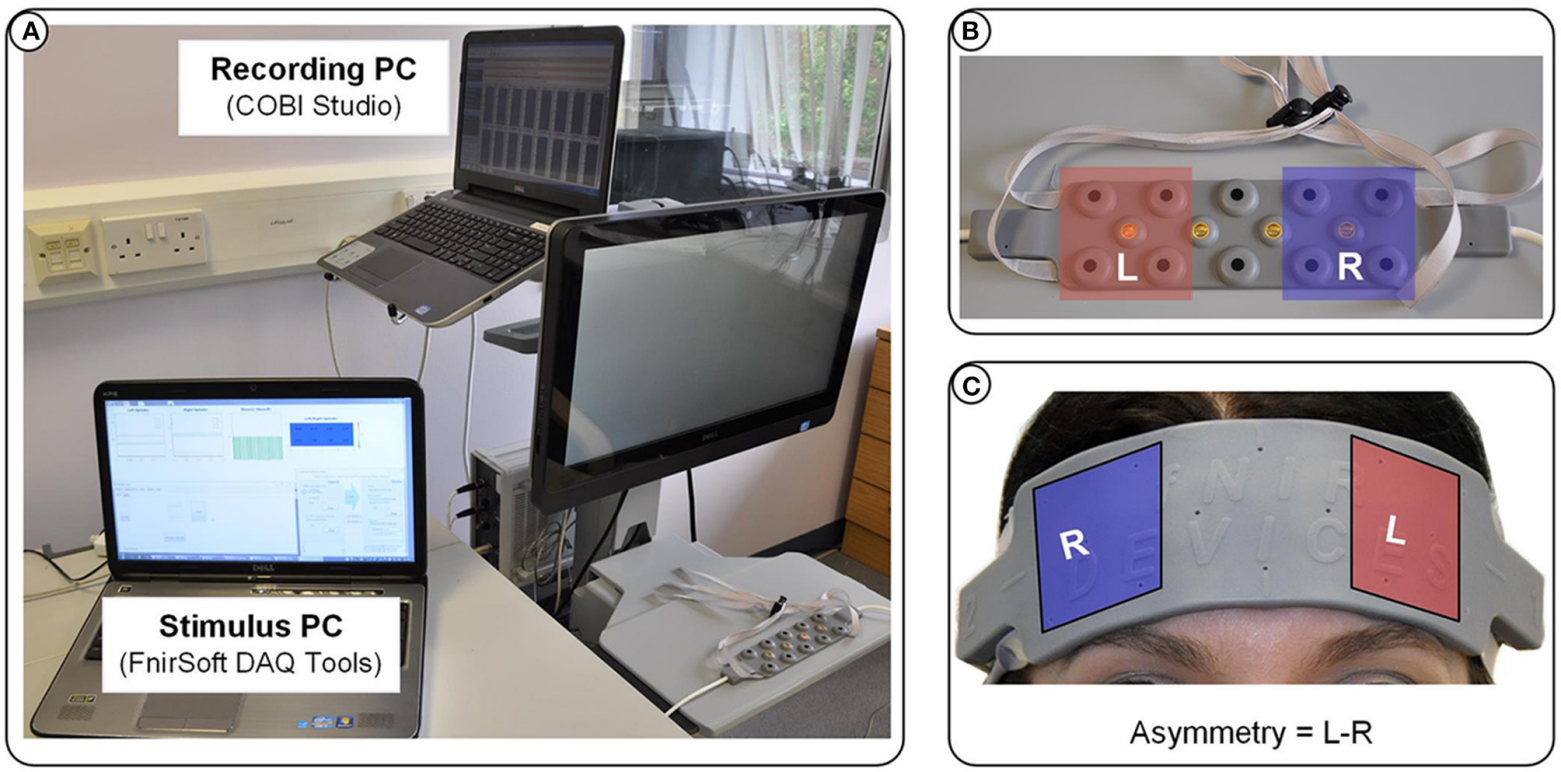
**(A)** Overview of the apparatus used in our experiments: fNIR Optical Brain Imaging System (fNIR400) by Biopac Systems with one PC dedicated to the data acquisition, and one PC dedicated to running the simulation and visualization of the stimulus presented in real-time to the subjects. **(B)** The 16-channel sensor placed on the subjects' forehead **(C)** showing the selected channels for the calculation of the asymmetry values.

For the three experiments presented here, we used an fNIR Optical Brain Imaging System (fNIR400) by Biopac Systems for data acquisition. Raw fNIRS data and oxygenation values were collected with 2 Hz sampling rate (using COBI Studio and FnirSoft), and was sent to the bespoke experimental software over TCP/IP (using FnirSoft DAQ Tools) (Figure 2A). A 16-channel sensor with a fixed 2.5 cm source-detector separation was placed on the subjects' forehead. For real-time application we used measurements of changes in HbO concentration, as opposed to deoxygenated or total hemoglobin (HbR and HbT, respectively) (Figures 2B,C). Values of HbO concentration changes were averaged over the four leftmost and four rightmost channels (located over the left and right DLPFC, respectively) to derive a simple metric of inter-hemispheric difference in the level of HbO change that could account for left prefrontal asymmetry (i.e., *Asymmetry* = *L*−*R*).

A bespoke graphical user interface was developed using C# and Windows WPF for each of the experiment, which includes a real-time visualization of HbO changes and asymmetry values. All these variables are logged during the experiment to facilitate the post-processing of the collected data. This graphical user interface was also used to manage the running of the experiment itself, which included displaying the required epochs (text, image, video, or more complex visualization) and managing their duration and the synchronization of all the software components. Finally, this interface was also used to implement visual feedback.

For the positioning of the different devices in relation to the subjects, we followed the recommendations of Solovey et al. (2009) regarding the use of fNIRS in a HCI setting. Subjects were seated ~47″ (120 cm) from a 24″ flat monitor in a dimly-lit, quiet (but not soundproof) room in a comfortable chair to minimize movements, with the fNIRS probe positioned over their forehead and covered with non-transparent fabric to prevent ambient light reaching the sensors. Subjects were instructed to refrain from moving their limbs, frowning and talking during data collection blocks.

### 3.2. Anger-Based NF—[ANG]

#### 3.2.1. Subjects

This experiment (Aranyi et al., 2015b) was conducted with twelve English-speaking adult subjects originally, though one subject had to be excluded due to technical problems. Thus, the effective sample size was eleven subjects (five females, mean age = 33.55 years, SD = 11.53, range: [24; 59]). Subjects had no history of psychiatric conditions and were right-handed. They all provided written consent prior to participation.

#### 3.2.2. Protocol

**Figure 3:**
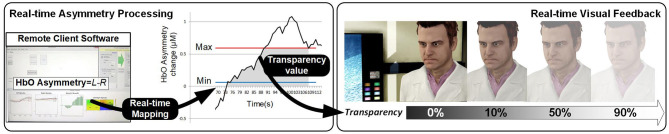
Experimental setup for the ANG experiment (see Figure 1 [ANG] for overall visual feedback setup).

**Figure 4:**
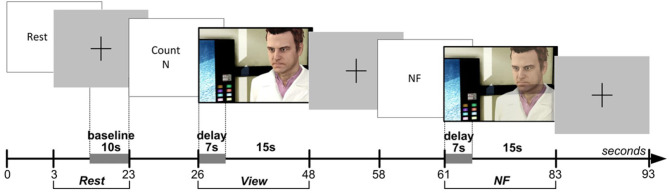
Protocol design for the ANG experiment.

Full details of the protocol design can be found in Aranyi et al. (2015b), but we will outline the essential details here (Figure 3 for the details of the overall setup). Subjects were instructed that they would go through a sequence of blocks, each comprising three main epochs: Rest, View and NF (Figure 4). During Rest, the baseline for calculating HbO data is acquired for the block as a whole. The View epochs correspond to control conditions, during which the subjects watch an idle animation of the character while given a cognitive task, *counting*, that keeps them in a neutral state, hence providing a reference for prefrontal asymmetry levels. Finally, the NF epochs consist in subjects expressing anger toward the character and receiving visual feedback. Each subject completed two training blocks to get acquainted to the task, followed by six blocks for the experiment itself, during which HbO asymmetry was monitored and recorded.

#### 3.2.3. Results

**Figure 5:**
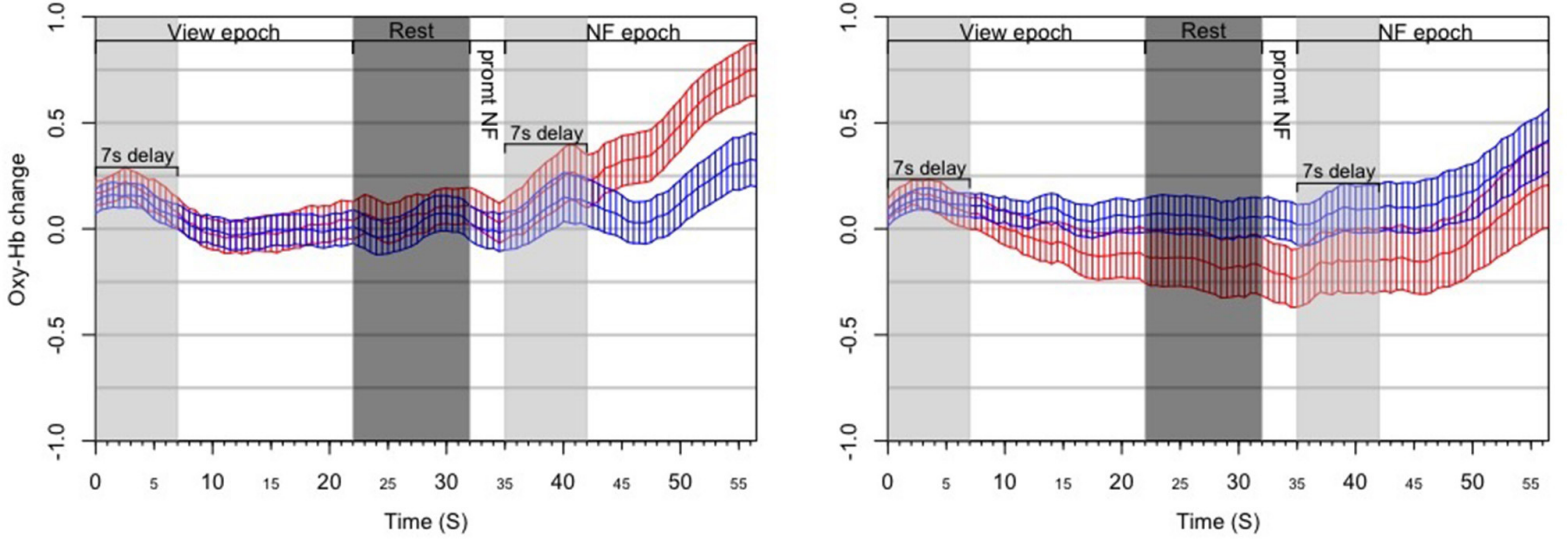
Results from the *post-hoc* analysis of the ANG experiment, illustrating the dynamics of PFC asymmetry over the whole experiment for *successful* blocks **(Left)** and *unsuccessful* blocks **(Right)** successful blocks demonstrates a significant increase of the left-side oxygenation compared to unsuccessful blocks.

We treated a block as successful if the mean of asymmetry values during the NF epoch was statistically significantly larger than the mean of asymmetry values during the View epoch within the same block (Figure 5). As opposed to simply comparing asymmetry scores during NF to the overall baseline, we compared asymmetry scores between the successive View and NF epochs because the visual stimulus was very similar (and conceptually the same) in the two epochs. Moreover, we set conditions for controlling subjects' cognitive activity during these epochs (counting during View and expressing anger during NF), whereas thought processes during Rest were not controlled. Thus, the View epoch served as a control condition within each block. Because the hemodynamic response measured by fNIRS occurs in ~7 s, we discarded the first 7 s of data in each View and NF epoch for determining block success. The system determined block success by performing an independent *t*-test on the set of asymmetry scores collected during successive View and NF epochs within a block. In particular, it calculated mean and standard deviation of asymmetry scores in both epochs, and then calculated the t value. Since removing the first 7 s left 15 s of data per epoch (at least 29 data points sampled at 2 Hz), the software used the t critical value of 2.05 with 28 degrees of freedom for *p* (two-tailed) = 0.05 as a threshold for success. Furthermore, to quantify the extent of block success by expressing the distance of the distribution of asymmetry scores during successive View and NF epochs, the experimental software calculated the Cohen's *d* effect-size measure, which is the difference between two means divided by the pooled standard deviation. This way we characterized each block with a dichotomous success value (success/fail) and a continuous success score (Cohen's *d* or *d* for short) that reflects the distance between the distribution of asymmetry values between View and NF epochs within the same block.

### 3.3. Virtual Agent—[RAP]

#### 3.3.1. Subjects

This experiment (Aranyi et al., 2016) was conducted with eighteen English-speaking adult subjects, though data from one subject was discarded due to technical problems during data collection. Thus, the effective sample size was seventeen subjects (eight females, mean age = 35.11 years, SD = 11.25, range: [21; 60]). Subjects were right-handed and were not treated for psychiatric conditions.

#### 3.3.2. Protocol

**Figure 6:**
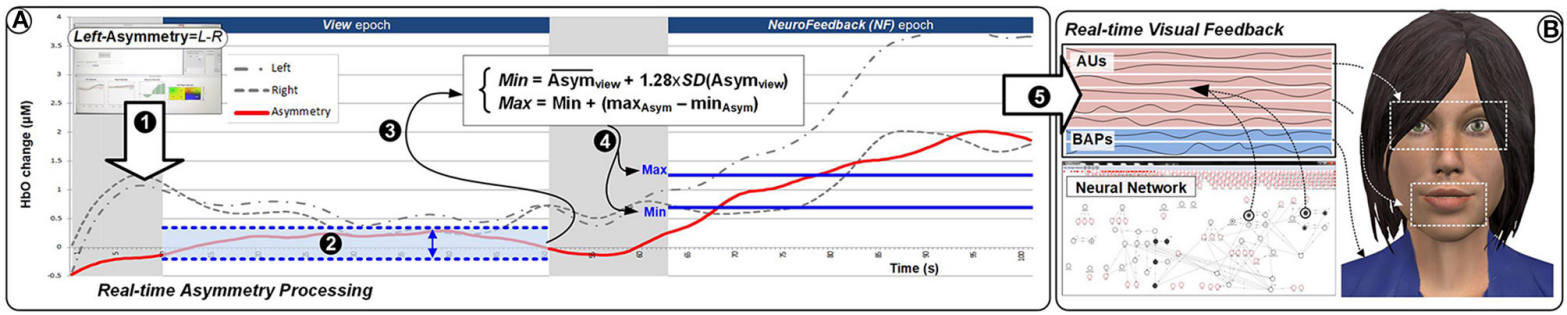
**(A,B)** Experimental setup for the RAP experiment (see Figure 1 [RAP] for overall visual feedback setup).

**Figure 7:**
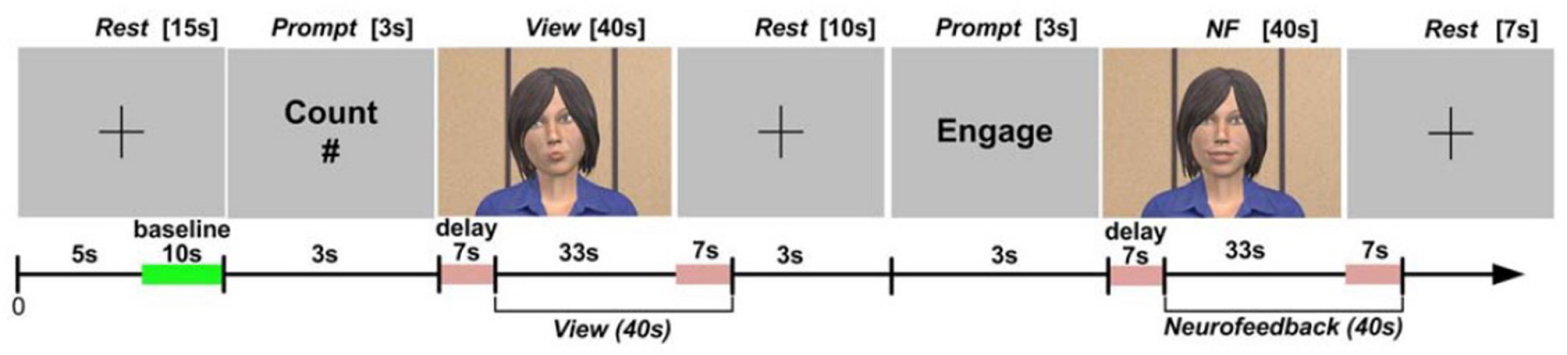
Protocol design for the RAP experiment.

Figure 6 provides an overview of the details of the overall setup. The experimental task consisted in completing eight identical blocks (preceded by a practice block which was not analyzed). The structure of the blocks is presented in Figure 7. Each block included three epochs: Rest, View, and Engage. During Rest epochs, subjects were instructed to look at a crosshair located in the center of a gray screen to try to clear their head of thoughts and relax. During View epochs, subjects were instructed to keep looking at the agent while carrying out a simple mental counting task (counting backwards from 500 by increments of a given integer). This task was included to control for unwanted mental processes. During Engage epochs, subjects were instructed to engage with the ECA through positive thinking, and to “cheer her up” with their thoughts. We were deliberately vague with support instructions in order to allow subjects to develop their own cognitive strategies. After completing each block, subjects were asked to describe their strategies in general terms. During Engage epochs, subjects received real-time feedback of their left-asymmetry. To ensure consistent mapping of individual variations in left-asymmetry onto the feedback signal, we used the range of variation of HbO asymmetry during the View epoch in each block to determine the mapping of the level of engagement from the user to the visual feedback signal. This was calculated by the experimental software during the last 3 s of the Rest epoch between the View and Engage epochs. We defined the minimum point for mapping Min as the mean of left-asymmetry values during the View epoch plus 1.28 times their standard deviation. In normally distributed asymmetry scores, this threshold would result in no feedback for 90% of the spontaneous asymmetry variations during the reference (View) epoch. To determine the maximum Max point for mapping, we increased the threshold asymmetry value for feedback Min by the variation range of asymmetry values during the View epoch. Asymmetry values within the range [Min; Max] during the Engage epoch were mapped linearly onto the ECA's facial expression, with the same 2Hz frequency as the acquisition of asymmetry values.

#### 3.3.3. Results

Subjects were instructed to refrain from talking, frowning and moving their limbs during fNIRS data collection periods within the protocol. Additionally, we applied a sliding-window motion artifact rejection (SMAR) to each channel used for calculating the asymmetry metric which was inspected *post-hoc* to identify motion artifacts during NF. For *post-hoc* analyses, raw data were low-pass filtered using a finite impulse response filter with order 20 and 0.1 Hz cut-off frequency (Ayaz et al., 2010). For this experiment (Aranyi et al., 2016), we used a sliding-window motion artifact rejection (SMAR) procedure, which rejected motion-affected periods in the fNIRS signal. This was an experiment which had greater potential for upper body movement as the subject could try to align to the agent non-verbal behavior which also included head and upper body motion.

**Figure 8:**
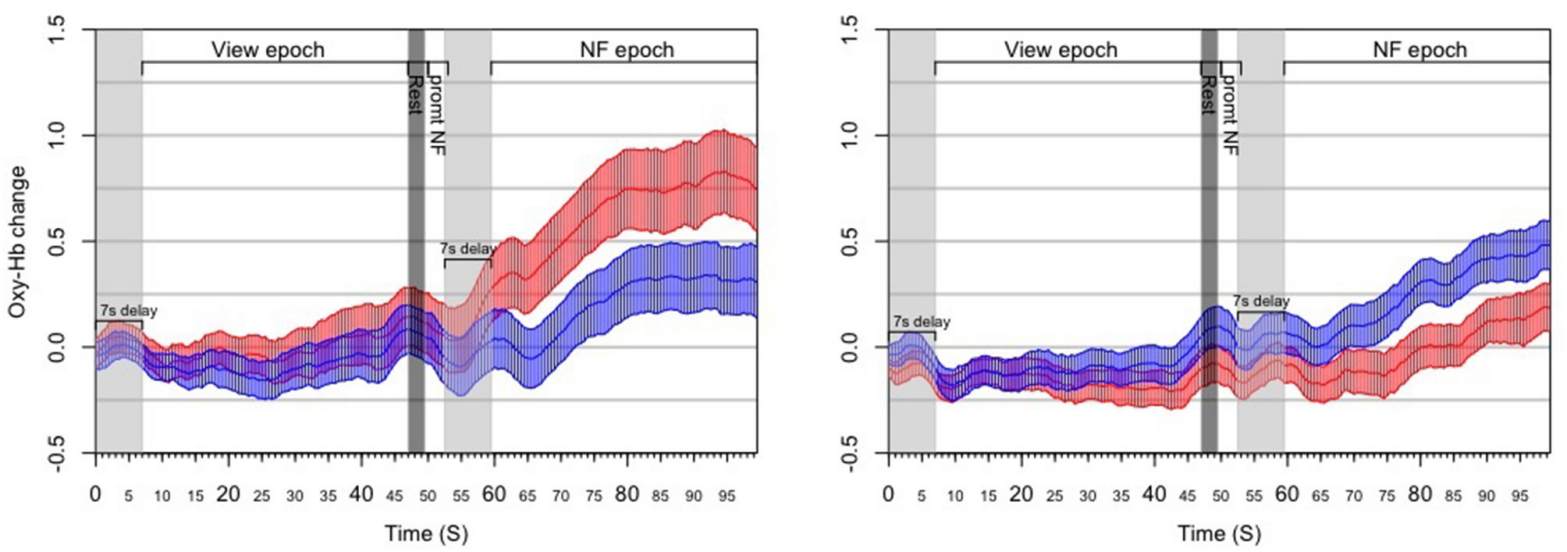
Results from the *post-hoc* analysis of the RAP experiment, illustrating the dynamics of PFC asymmetry over the whole experiment for *successful* blocks **(Left)** and *unsuccessful* blocks **(Right)** successful blocks demonstrates a significant increase of the left-side oxygenation compared to unsuccessful blocks.

Moreover, we have applied a counting task during reference epochs rather than passive visualization: while counting tasks are known to activate the PFC (Barth et al., 2016) they are also neutral toward affective and motivational aspects, which allows us to claim greater specificity for measuring NF activation compared to our reference epoch (Figure 8).

### 3.4. Motivational BCI—[HEU]

#### 3.4.1. Subjects

This experiment (Cavazza et al., 2017) was conducted with eleven adults (three females; mean age = 37.18 years, SD = 11.21, range = [20; 52]) who were right-handed, reported no treatment history for psychiatric conditions and provided written consent prior to participation. Subjects were seated in a dimly-lit room in a comfortable chair to minimize movements, with the fNIRS probe positioned over their forehead and covered with non-transparent fabric to prevent ambient light reaching the sensors.

#### 3.4.2. Protocol

**Figure 9:**
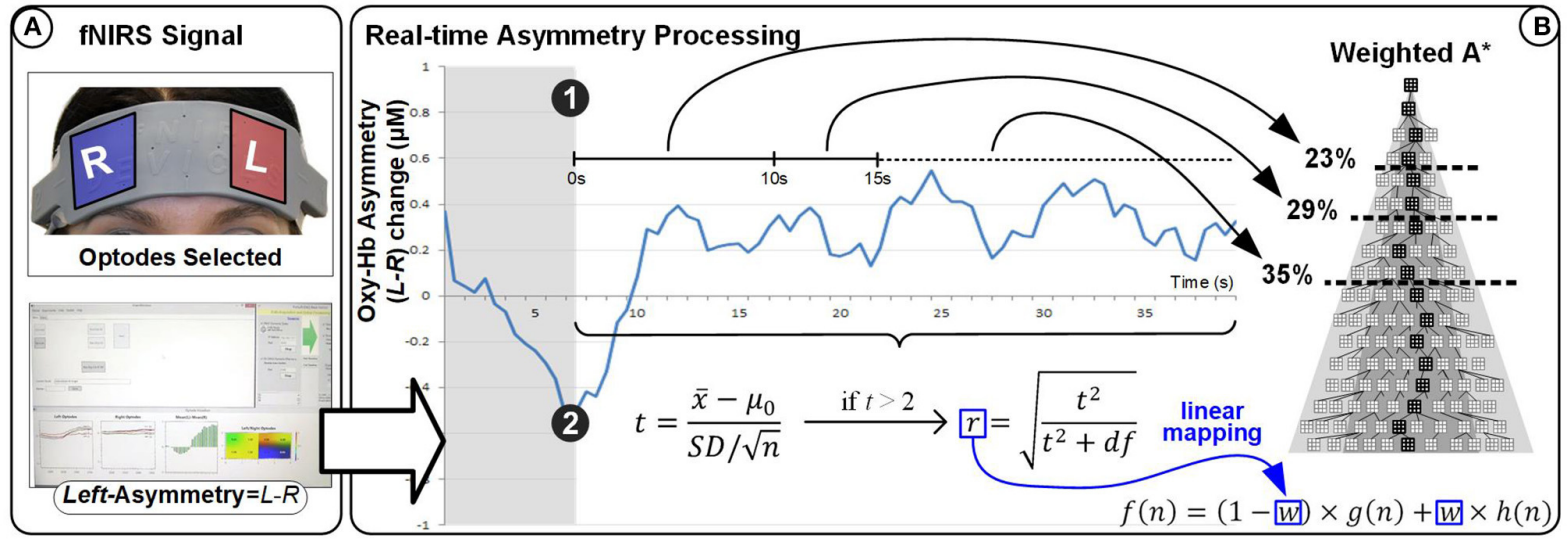
Experimental setup for the HEU experiment (see Figure 1 [HEU] for overall visual feedback setup). A* represent is artificial intelligence search algorithm.

**Figure 10:**
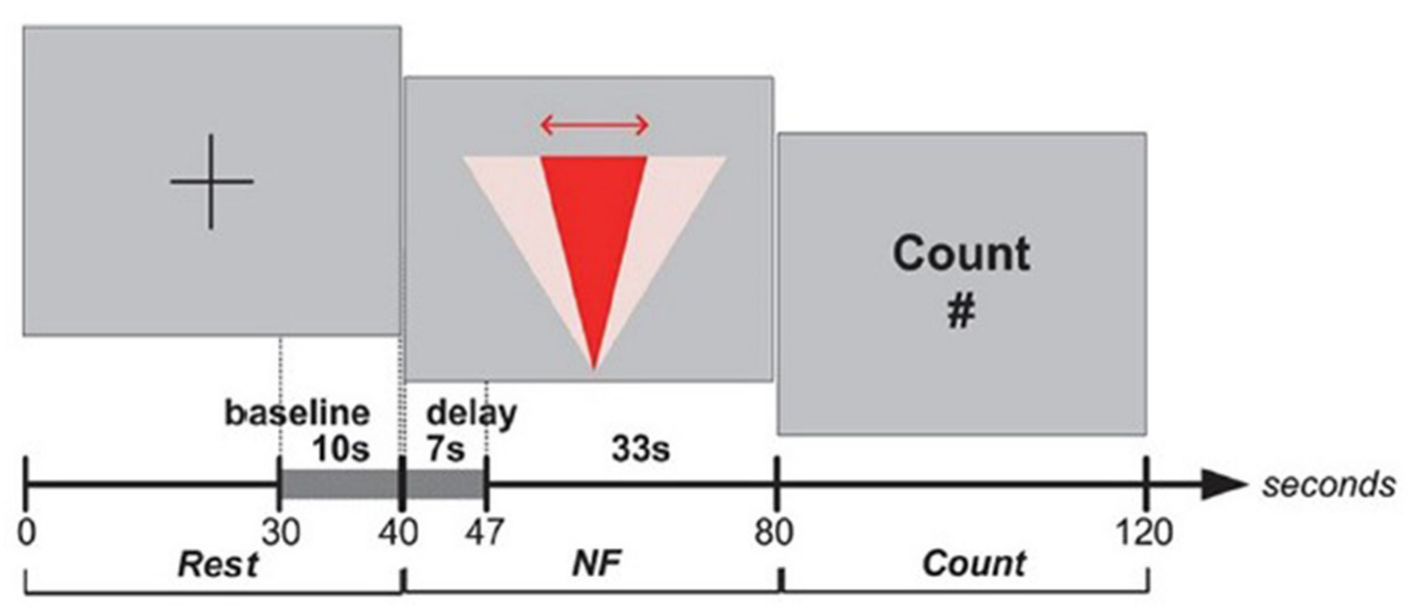
Protocol design for the HEU experiment.

HbO values were averaged over the four leftmost and the four rightmost channels (located over the left and right dorsolateral prefrontal cortex, respectively). Average right HbO was subtracted from average left HbO to derive a simple, real-time prefrontal asymmetry score rejecting differential changes in oxygenation. We developed bespoke experimental software for generating real-time feedback and interfacing with the WA* algorithm (Figure 9). Response time is an important component of NF systems; however, (Zotev et al., 2016) reported successful fMRI-based NF despite the ~7 s delay of the BOLD signal. Since delay using fNIRS is comparable, we sought inspiration from the experimental protocol of (Zotev et al., 2016). The overall protocol design for the experiment is described in Cavazza et al. (2017) and in Figure 10.

#### 3.4.3. Results

**Figure 11:**
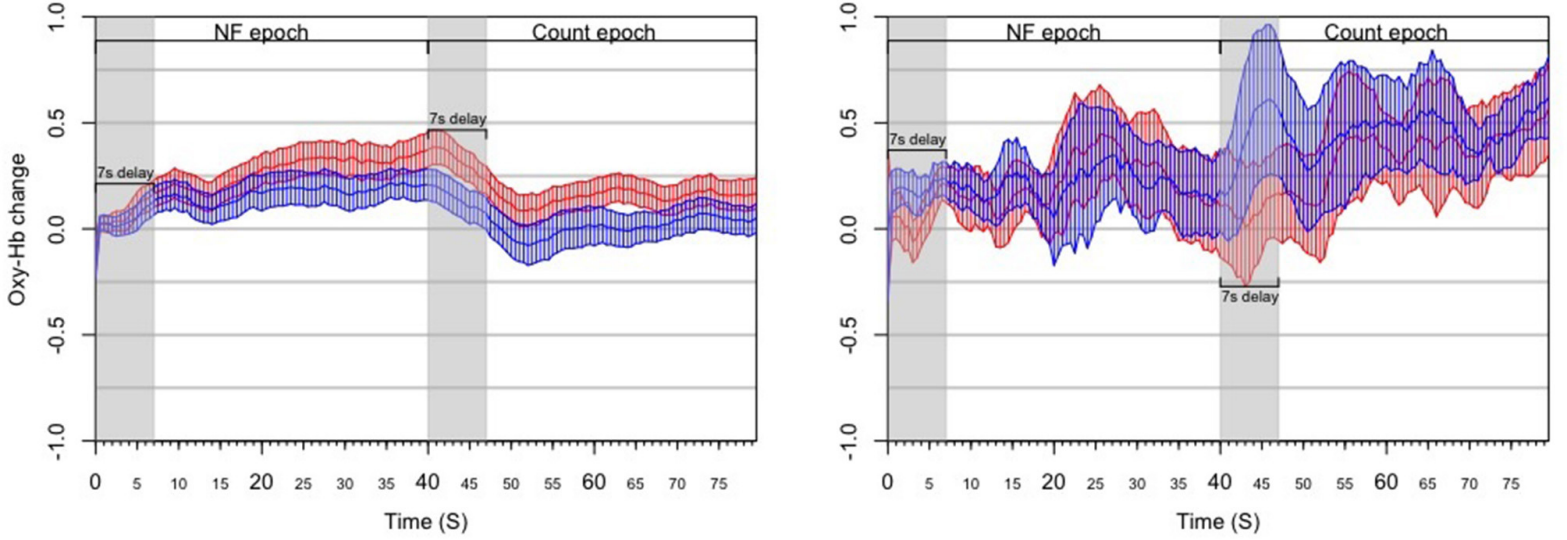
Results from the *post-hoc* analysis of the HEU experiment, illustrating the dynamics of PFC asymmetry over the whole experiment for *successful* blocks **(Left)** and *unsuccessful* blocks **(Right)** successful blocks demonstrates a significant increase of the left-side oxygenation compared to unsuccessful blocks.

Out of all 66 blocks completed by the eleven subjects, 38 (58%) contained an NF epoch with statistically significant left-side asymmetry; these blocks were considered successful. Each subject had at least one successful block, and eight subjects (73%) had at least three successful blocks (i.e., half of blocks successful). No subject achieved NF success on all six blocks. Since fNIRS signals are relative values, it can be difficult to compare them across subjects (Sakatani et al., 2013); moreover, the magnitude of oxygenation changes can also differ substantially across blocks within the same subject (Figure 11). Our mapping strategy was designed to mitigate the issue of comparability.

## 4. Data Formats and Datasets

### 4.1. Data Formats

The benefits of offering datasets to the BCI community must allow for the data to be easily manageable by all, which includes requirements, such as the ability to process the data with a wide range of modern software, in our case this includes Matlab or R, and also the ability to account for latest offerings in terms of programming languages, such as Python or Julia.

Other initiatives have supported the exchange of NIRS data, for instance the Shared Near Infrared Spectroscopy Format (SNIRF)^4^, developed by the Society for functional Near Infrared Spectroscopy. In an effort to facilitate the sharing of NIRS data, they have developed the Shared Near Infrared Spectroscopy Format (SNIRF). It follows a hierarchical data format—HDF5 which is a general purpose, machine-independent standard for storing scientific data in files, developed by the National Center for Supercomputing Applications (NCSA). As well as SNIRF, they have also developed two other text-based alternatives for platforms that do not support HDF5—JNIRS and BNIRS which are JSON and binary JSON files with the forementioned file extensions.

We have opted for a more portable and lightweight alternative to HDF5: JSON. To facilitate access to our data, we also provide binary data files for the most commonly used scientific languages—R, MATLAB, Python and Julia. The binary data allows for instant access to the data without the prerequisite of being familiar with HDF5 or JSON and acquainted with the necessary libraries that are needed to load the data in any given environment.

**Figure 12:**
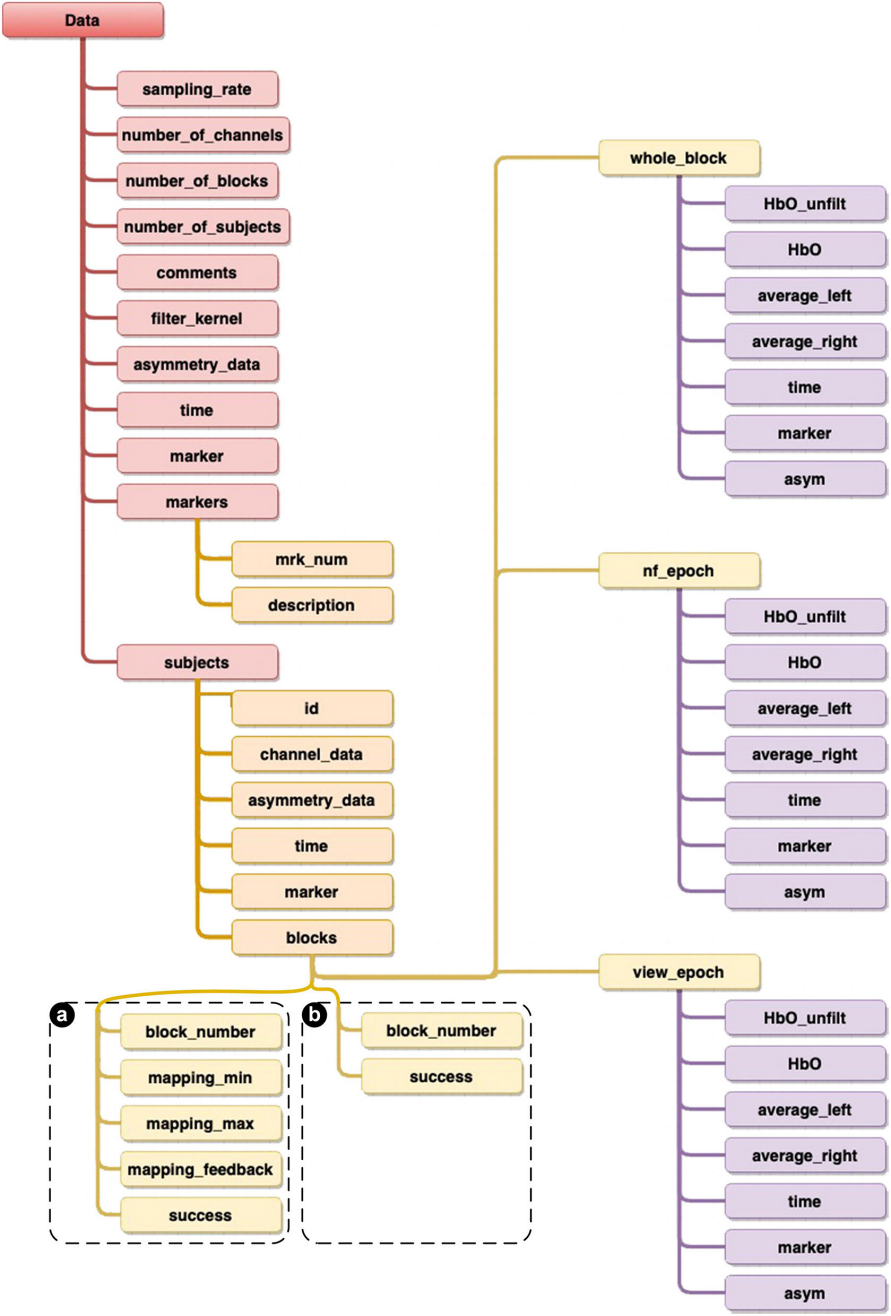
Diagram providing details of the structure of the overall datasets. The only difference in structures are shown in the blocks data, where **(a)** is specific to both [ANG] and [RAP] experiments, whilst **(b)** is specific to the [HEU] experiment.

Unlike SNIRF, which follows a generic filing-like hierarchical data structure, we have taken an object-oriented approach to structuring our data. This approach is self-contained and is descriptive of our experiments as we have objects that define a subject, an experimental block and an epoch (Figure 12). This approach facilitates the understanding and possible analysis of the data, as we have included as many properties as needed for ease of use and to accurately depict our experiments, such as a Boolean value property which indicates whether an experimental block was characterized as successful or not, the unfiltered HbO values for each channel used and the filtered asymmetry scores used during the NF epoch—and many more.

Our intention was to provide datasets ready for use, i.e., requiring minimum data wrangling prior to analysis. Though SNIRF provides a generic mechanism to share NIRS data, one would still need to extract relevant information and restructure the data depending on the intended data analysis. Our data format also reflects the fact that we are sharing fNIRS data in a NF context, and supports additional annotations typical of NF on top of RoI signal dynamics (in this case, PFC activity).

Thus, we decided to provide our datasets in multiple file formats so as to expand the usability of our datasets across software and languages—we therefore provide our data as files for MatLab (file extension *.mat*), R (file extension *.RData*), JSON (file extension *.json*), Pickle (file extension *.pckl*), and Julia (file extension *.jld*).

In this section, we provide details of the overall structure of the datasets, as well as highlighting detailing specificities, in order to make them as accessible as possible for further processing and analysis using programming languages Python or Julia, or from recognized data processing and analysis packages, such as Matlab and R. We first present generic data which are common to all our experiments, then we provide details about the specific Subjects data, as well as Blocks and Epochs, and finishing with a short discussion on Time Series (see framework and structural datasets details in Figure 12, as well as a summary of both the experiments settings in Table 1 and participants' demographic information in Table 2).

**Table 1:** Comparison table of overall protocol and analysis settings for the three experiments.

**Experiment**	**HEU**	**ANG**	**RAP**
Threshold	0	Dynamic (M + 1.28*SD)	Dynamic (M + 1.28*SD)
Maximum	1.1 (fixed)	Dynamic (min + range)	Dynamic (min + range)
Practice	3 blocks	2 blocks	1 block
N blocks	6	6	8
Baseline task	Rest	Rest	Rest
Reference epoch	No	Yes	Yes
Test	Parametric	Parametric	Bootstrapping
Success test	Real-time	Real-time	*Post-hoc*
Filtering	No	No	Yes (FIR, SMAR, detrending)
Success measure	r	d	r
Delay treatment	Remove 7 s	Remove 7 s	Windowing
Effective NF epoch length	33 s	15 s (+15 s reference task)	40 s (+40 s reference task)
N subjects	11	11	17
Block success	58%	58%	58%
Subject success	73%	73%	70%

**Table 2:** Comparison table of participants' demographic information for the three experiments.

**Experiment**	**HEU**	**ANG**	**RAP**
N subjects	11	11	17
N female subjects	3	5	8
Mean age	37.18	33.55	35.11
SD age	11.21	11.53	11.25
Range	[20; 52]	[24; 59]	[21; 60]

### 4.2. Generic Data

#### 4.2.1. Sampling Rate and Number of Channels

By default, our fNIR system records two wavelengths and dark current for each 16 optodes, totalling 48 measurements for each sampling period. Although the sampling rate of the latest generation systems can be up to 10 Hz, our experiments were recorded at 2Hz sampling rate (*Sampling_rate*). Although the fNIR system provides full data for all 16 optodes, as we only consider data for the calculation of the asymmetry scores, we here provide HbO data for eight optodes (*Number_of_channels*). Asymmetry calculations are derived from the four leftmost and four rightmost optodes, as a difference of Left minus Right (see Figures 2B,C).

#### 4.2.2. Asymmetry Data and Filtering

The asymmetry data (*Asymmetry_data*) calculated in real-time during the experiments is provided in the datasets as a tensor in the following format (subject, time, blocks). This subset constitutes the asymmetry data for all subjects and all blocks in the experiment considered. Each epoch within has been resampled and the data has been filtered using FIR filter kernel (*Filter_kernel*) applied as a low-pass filter to each raw channel data.

#### 4.2.3. Time and Blocks

All data provided includes timestamps in the form of a normalized time vector (*Time*) which is the temporal reference for all data in the experimental blocks considered. As described in detail for each protocol design in the previous section, we also record the number of experimental blocks for each subject (*Number_of_blocks*). These individual subject blocks also include the practice blocks. Practice blocks were actual blocks, as per the experimental design, used as a prior task for the subjects to acquaint themselves with the task which was expected from them. Subjects were not directly influenced by the experimenter during these blocks, but were given the opportunity to discover what the realtime system consisted of, overall. Although, practice blocks were also logged in our original data, we do not include them here, since they were not part of our original analysis.

#### 4.2.4. Markers

All our experiments include the very important markers data (*Markers*), which has been set consistently across our three experiments. It is provided as a collection of marker IDs, which include the “num,” being the marker number used to label the time series, as well as a “description” providing details of the epoch they refer to (e.g., 54 is “NF epoch”). These marker IDs are generated during the experiments so as to facilitate the extraction of the blocks from the raw data for the post-analysis. Marker data (*Marker*) is provided as a vector of the markers for each data point, so as to be able to label individual data points with the allocated reference marker, which then provides the details of which epoch they refer to.

### 4.3. Subjects

The all-important data recorded per subject is being made available on a per subject basis. For each subject we provide a reference (*id*) which is the subject's unique identifier. This identifier was generated following the convention “MDDN,” where M is the number of the month of the day the experiment took place, DD is the day of the experiment, and N is the subject order on the day of the experiment—(i.e., 3,241 is the 1st subject to take part in the experiment on the 24th of March. This method of anonymizing the subjects' details were deemed sufficient to be able to retrieve the subject's data if they had decided, at any point, to retract their data from the experiment analysis. (*Channel_data*) is a tensor in the format (channel, time, blocks) containing the channels for all blocks. Each epoch within has been resampled and the data has been filtered. The asymmetry information provided (*Asymmetry_data*) is a matrix in the format (time x blocks) containing the asymmetry data for all blocks. Each epoch within has been resampled and the data has been filtered. As expected from any data information, time stamps are also provided for each data entry (*Time*), which is a normalized time vector providing temporal labeling for the “Channel data” and “Asymmetry data” time series. Then, as presented previously, we also include marker data (*Marker*) as a vector of markers for segmenting the “Channel data” and “Asymmetry data” time series. And finally, (*Blocks*) is a collection of all blocks data. This is presented in the next section.

### 4.4. Blocks

Blocks are characterized by their identifier (*Block_number*) which describes the order of the block (starting at zero). We define a boolean variable (*Success*) indicating whether the block was successful based on the actual success criteria defined for the considered experiment (refer to the above sections for details of the definition of the success criteria). (*Whole_block*) is a variable which contains the data for the whole block (details are provided in the next section on Epochs). As we have presented in the details of the experiments above, the two important epochs are provided here as View (*View_epoch*), being a variable which provides the details of the View epoch data only, after having been segmented from the *Whole_block*, and the NF epoch data (*NF_epoch*), being a variable which provides the details of the NF epoch data only, after having been segmented from the *Whole_block*. Contrary to the HEU experiment, in both the ANG and RAP experiments, mapping is defined between the realtime asymmetry value processed and the feedback value, which is calculated on the basis of a [Min; Max] range—determined in realtime from the reference View epoch. Thus these two experiment datasets also include the following information for each block:

- (*Mapping_min*) is the value calculated as the lower bound value as: the Mean of the View epoch + 1.28 standard deviation of View epoch.
- (*Mapping_max*) is the value calculated as the upper bound value as: the Mapping min value + range of the View epoch.
- (*Mapping_feedback*) is the actual NF signal value mapped to the feedback.

### 4.5. Epochs

After having described the details of the actual structure of the data presented in the blocks, we are providing details for the epoch data points. We note that *Whole_block, View_epoch*, and *NF_epoch* follow the same data structure. (*Time*) is the un-normalized time vector containing the exact time each data point was recorded during the experiment. This provides accurate and detailed overview of the actual recordings and affords possibilities for the potential further analysis. (*Marker*) is a vector of markers allowing for the segmentation of time, HbO filtered and unfiltered, Average left, Average right, and asymmetry values. (*HbO*) provides a matrix of the low-pass filtered HbO channel data in the format channel x time. (*HbO_unfiltered*) provides the same matrix format for the unfiltered HbO channel data. (*Average_left*) is the average value of the four leftmost optodes. (*Average_right*) is the average value of the four rightmost optodes. And finally, (*Asymmetry*) is the actual asymmetry value generated as the difference of *Average_left* and *Average_right*.

### 4.6. Time Series Discussion

The ANG dataset differentiates between approach and valence, albeit not perfectly, and could be used to experiment whether differences of magnitude take place by removing the valence component. In the HEU dataset, NF success above baseline is used primarily as a trigger so could be of interest on comparative study of NF dynamics but perhaps less on NF epoch-based validation. The RAP dataset is closer to previous EEG experiments (Cavazza et al., 2014) and the one with perhaps the most potential for confounding various aspects of PFC asymmetry in terms of its dimensional interpretation (approach, valence). On the other hand, it has some of the longest fNIRS NF epochs (Kohl et al., 2020) and is a good candidate to study signal dynamics.

## 5. Conclusions

We have described three datasets for fNIRS PFC asymmetry, which correspond to one of the most investigated signals in social and affective neuroscience and also one of the main areas for fNIRS NF. Although the focus of our BCI experiments were primarily on the motivational dimension, the DLPFC signals can also be of interest to researchers requiring comparative data when investigating cognitive workload or other dimensions, such as valence. As these datasets cover different NF variants, they should be valuable to investigate signal dynamics across epochs of different lengths as well as issues around baselining and reference epochs. Since they all have been previously analyzed as part of various publications (Aranyi et al., 2015b, 2016; Cavazza et al., 2017), they can also support experiments with various statistical methods for *post-hoc* epoch validation. We have endeavored to facilitate this through the various formats we have embedded data into, which should support various processing pipelines in data analysis or machine learning.

## Data Availability Statement

The datasets generated for these studies can be found in the BCI NeuroFeedback fNIRS repository: https://github.com/fcharles-BU/BCI-NF-fNIRS.

## Ethics Statement

The studies involving human participants were reviewed and approved by Teesside University Research Ethics Committee, Middlesbrough, Tees Valley, TS1 3BX, UK. The patients/participants provided their written informed consent to participate in this study.

## Author Contributions

FC contributed to the data collection, systems development, and writing of the manuscript. CD contributed to the dataset generation and analysis, and writing of the manuscript. MC contributed to the original experimental designs and writing of the manuscript. All authors have made direct and substantial intellectual contributions to the article and approved it for publication.

## Conflict of Interest

The authors declare that the research was conducted in the absence of any commercial or financial relationships that could be construed as a potential conflict of interest.

## Footnotes

^1^We are primarily considering here NF systems for which there is an element of regional specificity or spatial resolution, which is generally the case for fNIRS and fMRI-based NF.

^2^The following list includes NF experiments based on hemodynamic signals, fMRI or fNIRS.

^3^This review already includes one of our own experiments corresponding to the RAP dataset.

^4^https://github.com/fangq/snirf/blob/master/snirf_specification.md
